# Mediterranean herb extracts inhibit microbial growth of representative oral microorganisms and biofilm formation of *Streptococcus mutans*

**DOI:** 10.1371/journal.pone.0207574

**Published:** 2018-12-12

**Authors:** Joachim Hickl, Aikaterini Argyropoulou, Maria Eleni Sakavitsi, Maria Halabalaki, Ali Al-Ahmad, Elmar Hellwig, Nektarios Aligiannis, Alexios Leandros Skaltsounis, Annette Wittmer, Kirstin Vach, Lamprini Karygianni

**Affiliations:** 1 Department of Operative Dentistry and Periodontology, Medical Center, University of Freiburg, Faculty of Medicine, University of Freiburg, Freiburg, Germany; 2 Department of Pharmacognosy and Natural Products Chemistry, Faculty of Pharmacy, National and Kapodistrian University of Athens, Athens, Greece; 3 Institute of Medical Microbiology and Hygiene, Faculty of Medicine, University of Freiburg, Freiburg, Germany; 4 Institute for Medical Biometry and Statistics, Faculty of Medicine and Medical Center, University of Freiburg, Freiburg, Germany; 5 Clinic for Preventive Dentistry, Periodontology and Cariology, Center of Dental Medicine University of Zurich, Zurich, Switzerland; Tallinn University of Technology, ESTONIA

## Abstract

In light of the growing antibiotic resistance, the usage of plant-derived antimicrobial agents could serve as an effective alternative treatment against oral infections. The aim of this study was to investigate the antimicrobial and antibiofilm activity of Mediterranean herb extracts against representative oral microorganisms. The extraction procedures and the analysis of the obtained extracts were performed under established experimental conditions. The minimum inhibitory (MIC) and bactericidal (MBC) concentrations of the methanol extracts of *Cistus creticus* ssp. *creticus*, *Cistus monspeliensis*, *Origanum vulgare*, *Rosmarinus officinalis*, *Salvia sclarea* and *Thymus longicaulis* against eight typical oral bacteria and the fungus *Candida albicans* were determined. The antibiofilm activity against *Streptococcus mutans* was also quantified using the microtiter plate test. Overall, all tested extracts inhibited effectively the screened obligate anaerobic microorganisms and in concentrations ≥0.3 mg ml^-1^ had moderate to high antibiofilm activity comparable to that of chlorhexidine (CHX) against *S*. *mutans*. In particular, *R*. *officinalis* (MIC: 0.08–5.00 mg ml^-1^) and *S*. *sclarea* (MIC: 0.08–2.50 mg ml^-1^) showed the highest antibacterial activity, while *Cistus* spp., *R*. *officinalis* and *S*. *sclarea* significantly inhibited *S*. *mutans* biofilm formation at 0.60, 1.25 and 2.50 mg ml^-1^, respectively. *Porphyromonas gingivalis* and *Parvimonas micra* were high susceptible to *O*. *vulgare* (MIC = 0.30 mg ml^-1^), whereas *T*. *longicaulis* eradicated all oral bacteria (MBC: 0.15–2.50 mg ml^-1^). Nevertheless, *C*. *albicans* showed no sensitivity to the tested extracts. In conclusion, the tested plant extracts could serve as alternative natural antibacterial and antibiofilm components against oral infections.

## Introduction

The statement of Hippocrates “Natural forces are the true healers of disease” reflects the fact that thousands of years before any synthetic medicaments were known, nature had been widely considered as the only limitless source of healing components. The ultimate intention has always been to use natural products with favorable antimicrobial, anti-inflammatory and antitumoral properties, without side effects at the chosen concentration, for medicinal purposes. Recently, we have therefore seen diverse plant extracts as well as pure natural compounds as part of various treatment protocols in daily medicinal use [1–5]. Especially plant extracts originating from the Mediterranean area belong to the most frequently screened natural resources for application in medicine [6–8].

Although almost about 350,000 plant species have been characterized to date, thousands of unexplored plant species need to be studied for their chemical or biological profile and thus, their potential as a non-pharmacological intervention for diverse diseases [9]. Following the evolution of all living organisms, plants have nowadays developed their own molecular antimicrobial strategies to survive, by producing secondary metabolites with synergistic action such as small antimicrobial peptides, alkaloids, coumarins, flavonoids, phenols, phenolic acids, quinones, saponins, tannins and terpenoids [10, 11]. For instance, the two main components of *Origanum vulgare*, namely thymol and carvacrol, present synergistic activity against *Streptococcus mutans*, *Streptococcus mitis*, *Prevotella oris*, *Prevotella intermedia*, *Micrococcus luteus* and others [12, 13].

Surprisingly, according to a recent review by Sender *et al*. the ratio between bacterial and host cells in humans is approximately 1:1 [14]. In particular, the oral cavity hosts 700 to 1000 different bacterial species mostly situated on both soft and hard tissues, e.g. teeth, gingiva, tongue, cheeks and palate [15]. Thus, bacterial diseases in oral cavity are more likely polymicrobial infections which etiologically correlate with caries, pulpitis, gingivitis and periodontitis [16]. The complex oral microbial communities are mainly organized in biofilms. During the initial phase of oral biofilm formation, the salivary pellicle is mainly colonized among others by *Streptococcus* spp., which bind with their receptors to host proteins like α-amylase, immunoglobulins, fibronectin, lactoferrin and α_2_-macroglobulin [17, 18]. It was shown that *S*. *mutans* strains in biofilms are up to 70,000 times more acid tolerant than their planktonic counterparts [19].

Taking the constantly increasing ineffectiveness of antibiotics into consideration, the necessity to develop new efficient treatment strategies against oral diseases has raised interest in natural products. For instance, nosocomial infections caused by *Acinetobacter baumannii* need an alternative treatment as carbapenem resistances have raised. The plant *Scutellaria baicalensis* seems to have one promising compound named norwogonin which showed antibacterial effects against *A*. *baumannii* [20] and *Actinidia deliciosa* methanol extract reduced its production of bacterial biofilm components [21]. Because of their secondary metabolism, natural products such as plants yield chemical reactions with significant pharmaceutical effects on various microorganisms. As a result, numerous natural extracts or their main compounds have been screened all over the world, indicating that herbs can improve the oral health [22]. Various plant products such as coffee, grape, cranberry juice, tea (green-, cistus-, black tea) have been tested for their potential to treat caries, periodontitis and periimplantitis [23, 24]. Especially Mediterranean herbs seem to have a high efficacy against representative oral microorganisms [24, 25].

Thus, the goal of the present report was to examine the antimicrobial and antibiofilm activity of six Mediterranean natural herb extracts against representative oral microorganisms *in vitro*. More precisely, the methanol extracts of *Cistus creticus* ssp. *creticus*, *Cistus monspeliensis*, *Origanum vulgare*, *Rosmarinus officinalis*, *Salvia sclarea* and *Thymus longicaulis* were tested against eight typical oral pathogenic microorganisms and the fungus *Candida albicans*. Additionally, two reference strains, namely *Staphylococcus aureus* and *Escherichia coli*, residents on skin and intestinal mucosa, respectively, were investigated. The antibiofilm effect of the extracts against *Streptococcus mutans* was also examined. Hence, three antimicrobial assays were applied: the minimal inhibitory concentration (MIC) assay, the minimal bactericidal concentration (MBC) assay and the biofilm plate assay. The null hypothesis was assumed that the tested extracts have no significant antimicrobial and antibiofilm impact on the screened microbial species.

## Materials and methods

### Extraction process

Aerial plant parts of the six different plant species were collected from various places of the Greek periphery. *Cistus creticus* L. (Cistaceae, Lat.: 35^o^ 23′17.76″ N, Long.: 24^o^ 54′50.89″ E, Elevation: 181 m) and *Cistus monspeliensis* L. (Cistaceae, Lat.: 35^o^ 17′29.13″ N, Long.: 25^o^ 32′15.33″ E, Elevation: 63 m) were collected from Crete, *Origanum vulgare* L. (Lamiaceae, Lat.: 37^o^ 16′42.22″ N, Long.: 22^o^ 40′34.36″ E, Elevation: 599 m) from Peloponnese, *Rosmarinus officinalis* L. (Lamiaceae, Lat.: 37^o^ 58′07.98″ N, Long.: 23^o^ 47′11.34″ E, Elevation: 253 m) and *Thymus longicaulis* C. Presl. (Lamiaceae, Lat.: 38^o^ 11′02.26″ N, Long.: 23^o^ 15′19.86″ E, Elevation: 1361 m) from Attiki and *Salvia sclarea* L. (Lamiaceae, Lat.: 39^o^ 52′54.37″ N, Long.: 20^o^ 38′01.89″ E, Elevation: 449 m) from Epirus. The collection sites were on public land. The University of Athens, being a public Institute, reserves the right to collect a small amount of plants for research purposes. No permits or permissions were required to collect samples from these public land areas. A small amount was collected for the needs of the investigation and the plants were not endangered or protected species. A voucher specimen of each plant is deposited at the herbarium of the Department of Pharmacognosy and Natural Products Chemistry, Faculty of Pharmacy, National and Kapodistrian University of Athens under the following numbers: *C*. *creticus*—KL 057, *C*. *monspeliensis*—KL 060, *O*. *vulgare*—F 009, *R*. *officinalis*—KL 163, *T*. *longicaulis*—P-K 011, *S*. *sclarea*–In 023. Collected plants were grinded (SCIS, Allenwest-Eac ltd) into fine homogeneous powders and extracted with ultrasound assisted the extraction (UAE). An Elma S 100H (Elmasonic) instrument was used with methanol (100%) as extraction solvent for 15 min at room temperature and ratio plant per solvent 1/10 (w/v). Extraction procedure was repeated twice for each sample. Methanol was evaporated to dryness under reduced pressure using a rotary evaporator (Buchi Rotavapor R-200) at 40°C.

### High performance thin layer chromatography (HPTLC) analysis

A Camag HPTLC instrumental setup was used for generating the fingerprinting of the various extracts. Extracts’ solutions were prepared by dissolving 10 mg of each extract in 1 ml of methanol. The samples of the plant extracts were applied onto 20 × 10 cm TLC plates (silica gel 60, F254, Merck) using the Automatic TLC sampler (ATS4, CAMAG) under the control of the software platform VisionCats 2.3 (Camag) with the following standard settings: 6 tracks with 8 mm bands, 8 mm distance from the lower edge, 20 mm from the left and right edges, and 10.4 mm between the different tracks. The application volume of the samples was 8 μl. The plates were developed with an automatic development chamber (ADC2) using standard settings: 20 min chamber saturation with filter paper, 10 min of plate conditioning at 33% relative humidity (MgCl_2_) and 5 min of plate drying. Dichloromethane, methanol, water (70:30:4; v/v/v) and ethyl acetate, methanol, formic acid, water (50:10:7:1; v/v/v/v) were used as the mobile phases. Images at 254 nm and 366 nm were recorded on a Visualizer 2 Documentation System (CAMAG, Muttenz, Switzerland).

### UPLC-HRMS & HRMS/MS analysis

UPLC-HRMS/MS analysis was performed on an AQUITY system (Waters) connected to a LTQ-OrbitrapR XL hybrid mass spectrometer (Thermo Scientific) equipped with an electrospray ionization (ESI) source and operated in negative mode. A UPLC separation gradient was developed in order to efficiently resolve all compounds for a qualitative analysis. The flow rate was set at 0.4 ml min^-1^ and the solvent system was (A) water 0.1% formic acid and (B) acetonitrile. The elution program was: 2% B for 2 min; 100% B in 18 min; and hold for 2 min. After return back to 2% B in 1 min, column equilibration was performed for 4 min at the end of the run. The injection volume was set to 10 μl and samples were injected at 0.3 mg ml^-1^ in water-acetonitrile solution (1:1) on a Supelco Ascentis Express C18 (100 x 2.1 mm i.d, 2.7 μm particle size). The HRMS & HRMS/MS data were acquired in negative mode over 100–1000 m/z range. The MS profile was recorded in full scan mode (scan time = 1 micro scans and maximum inject time = 500 ms). The ESI conditions were as follow: capillary temperature 320°C; capillary voltage -40 V; tube lens -120 V; ESI voltage 2.7 kV. Nitrogen was used as sheath gas (40 Au) and auxiliary gas (8 Au). For the HRMS/MS acquisitions, a data-dependent method including the detection (full scan) and fragmentation of the 3 most intense peaks per scan was used. The mass resolving power was 30,000 for both levels and the normalised collision energy (CID) in the ion trap was set to 35.0% (q = 0.25) for the HRMS/MS experiments. Chromatographic and spectrometric features were used for identification of extracts constituents such as retention time (Rt), polarity, accurate m/z, proposed elemental composition (EC), ring double bond equivalent (RDBeq) values as well as HRMS/MS spectra and derived fragmentation motifs. The raw data were acquired and processed with XCalibur 2.2.4 software from Thermo Scientific.

### Bacterial and fungal strains

Ten bacterial strains and one *C*. *albicans* strain were selected. Eight of the tested bacterial strains and *C*. *albicans* are typical residents of the oral cavity. *S*. *aureus* and *E*. *coli*, in contrast, can be mainly detected on skin and within the intestinal flora, respectively, and served as reference species. Among the selected strains, *Streptococcus mutans* DSM (German Collection of Microorganisms and Cell Cultures) 20523, *Streptococcus sobrinus* DSM 20381, *Streptococcus oralis* ATCC 35037, *Enterococcus faecalis* ATCC 29212 *and S*. *aureus* ATCC 25923 are facultative anaerobic Gram-positive species, whereas *E*. *coli* ATCC 25922 is facultative anaerobic but it has a Gram-negative cell wall. *Porphyromonas gingivalis* W381, *Prevotella intermedia* MSP34 (clinical isolate), *Fusobacterium nucleatum* ATCC 25586 and *Parvimonas micra* ATCC 23195 are obligate anaerobic bacteria. The only used fungus *C*. *albicans* DSM 1386 grows both as yeast and filamentous cells. All bacterial and fungal strains were kindly supplied by the Division of Infectious Diseases and the Institute of Medical Microbiology and Hygiene, Faculty of Medicine, University of Freiburg. The microorganisms were deposited at -80°C in basic growth medium containing 15% (v/v) glycerol until their use as formerly described by Jones *et al*. [26].

### Determination of the minimum inhibitory concentration (MIC)

Firstly, an overnight culture of each bacterial and fungal strain was prepared according to the guidelines of Clinical and Laboratory Standards Institute (CLSI) [27, 28]. Each microorganism was plated on Columbia blood agar plates (CBA) or yeast-cysteine blood agar plates (HCB). Facultative anaerobic bacteria and *C*. *albicans* were incubated on CBA at 37°C and 5–10% CO_2_ atmosphere for 24 h. The anaerobic bacteria were plated on HCB at 37°C for 48 h (anaerobic chamber, Anaerocult, Merck Chemicals GmbH, Darmstadt, Germany). A 0.5 / 1 McFarland standard suspension was prepared in 0.9% saline (NaCl) for facultative anaerobic bacteria and *C*. *albicans*, respectively. For the microdilution assay, all facultative anaerobic strains and *C*. *albicans* were then 1:10 diluted in BBL Mueller Hinton II Broth (Cation-Adjusted) (MHB, BD, Heidelberg, Germany). The anaerobic bacteria were set in Wilkins-Chalgren broth (WCB) as a 0.5 McFarland standard suspension. As previously described (ISO 20776–1: 2006 [29]), the cell density of facultative anaerobic bacteria, fungi and obligate anaerobic bacteria should be about 5 x 10^5^, 5 x 10^4^ and 5 x 10^6^ colony forming units (CFU) per ml, respectively. Afterwards, appropriate volumes of the MHB / WCB microbial cultures were transferred into a 96-well microtiter-plate using a multi-channel pipette.

### Preparation of extracts and procedure for MIC testing

The produced natural plant extracts were dissolved in dimethyl sulfoxide (DMSO, Sigma-Aldrich Chemie GmbH, Steinheim, Germany) at a concentration of 100 mg ml^-1^. All extract solutions in DMSO were screened in a concentration series ranging from 10 mg ml^-1^ to 0.02 mg ml^-1^ at dilution levels starting from 10-fold to 5120-fold. Each well of the 96-well microtiter-plate had a total volume of 100 μl. In order to exclude potential antimicrobial effects of the DMSO residuals, a dilution series of DMSO was examined in parallel. Wells containing solely MHB / WCB and a dilution series of 0.1% chlorhexidine (CHX) served as negative and positive controls for bacterial growth, respectively. Additionally, wells containing MHB / WCB and the added microbial strain acted as growth controls. The possibility of contamination was minimized by using sterile MHB / WCB. Thereafter, *E*. *coli*, *S*. *aureus*, *E*. *faecalis* and *C*. *albicans* were incubated at 37°C for 18 h, the three streptococci strains at 37°C under 5–10% CO_2_ atmosphere for 24 h, while anaerobic bacteria were kept at 37°C for 48 h in anaerobic incubation bags (Anaerocult IS, Merck Chemicals GmbH, Darmstadt, Germany). All assays for each bacterial and fungal strain were performed at least in duplicate. The highest MIC values were taken into consideration in case the MIC values of a specific strain were not identical. If the deviation between the two rows showed more than two dilution levels the determination with this extract was repeated. MIC was defined as the lowest concentration of each natural plant extract at which visible inhibition of bacterial growth was induced. The inhibitory impact of DMSO was taken into consideration if bacterial growth was observed in the co-tested DMSO dilution series.

### Determination of the minimum bactericidal concentration (MBC)

The minimum bactericidal concentration (MBC) was also assessed according to the CLSI guidelines [27, 28]. After completion of the MIC assay, the 96-well microtiter-plates were further incubated for MBC testing. In brief, 10 μl from each well containing the tested plant extract concentration series were plated on CBA or HCB. In particular, *E*. *coli*, *S*. *aureus* and *E*. *faecalis* were plated on CBA at 37°C for 24 h, streptococci and *C*. *albicans* were incubated on CBA at 37°C and 5–10% CO_2_ atmosphere for two days. The obligate anaerobes were cultivated on HCB at 37°C for five days. To create anaerobic conditions an anaerobic jar and the Anaerocult. A gas generator system (Merck, Darmstadt, Germany) were used. Finally, the CFU were determined visually. The MBC was defined as the concentration at which a three Log decrease in bacterial growth (= 99.9% inhibition) was detected compared to the growth control.

### Biofilm plate assay

The biofilm formation test was performed as already described by Al-Ahmad *et al*. [30]. Initially, a bacterial strain of *S*. *mutans* R15-8 (clinical isolate) was cultivated overnight at 37°C under aerobic conditions with 5–10% CO_2_ in MHB (BD, Heidelberg, Germany) containing 1% saccharose (MHB-S). Polystyrene 96-well tissue-culture plates (Greiner bio-one, Frickenhausen, Germany) were then filled with 100 μl of MHB-S, including ten different concentrations (0.02–10 mg ml^-1^) of the investigated plant extracts, and 5 μl of the *S*. *mutans* overnight culture (10^8^ CFU ml^-1^) were added to each well. A dilution series of DMSO was examined in parallel. Wells containing solely MHB-S and a dilution series of 0.2% chlorhexidine (CHX) served as negative and positive controls for bacterial growth, respectively. Thereafter, the 96-well plates were incubated for 48 h at 37°C in an aerobic atmosphere with 5–10% CO_2_. The culture medium was then discarded, and the wells were washed three times with 300 ml phosphate-buffered saline (PBS, Life Technologies, Darmstadt, Germany) per plate to remove non-adherent bacteria. The plates were then air dried and stained with Carbol Gentian Violet solution (Carl Roth GmbH + Co. KG, Karlsruhe, Germany) for microscopy, containing 0.1-<0.25% methyl violet, for 10 min. Excess stain was removed by rinsing the plates with distilled water. Afterwards, the plates were dried for 10 min at 60°C and after dye resolubilization by adding 50 μl of absolute ethanol (99.9% v/v) for analysis (Merck Chemicals GmbH, Darmstadt, Germany) in each well, the optical density was finally measured at 595 nm using the Tecan Infinite 200 plate reader (Tecan, Crailsheim, Germany). All tests were conducted in quadruplicate and the mean values were determined. During the analysis, the antibiofilm effect of each extract on *S*. *mutans* was categorized in three different groups with the aid of two different cut-off values: no biofilm production or C1, moderate biofilm production or C2, and high biofilm production or C3. The low cut-off value was estimated by adding three standard deviations of the blank to the negative control. The high cut-off value resulted after the measurement of the low cut-off value for three times.

### Statistical analysis

For a descriptive data analysis, the means and standard deviations regarding the optical density (OD) were computed. A two-sample *t* test was used for pairwise group comparisons between the plant extracts and the controls, namely CHX (positive control) and DMSO (negative control). Scatter plots of the OD values reflecting the level of biofilm inhibition by the plant extracts (low, moderate, high) were finally graphically demonstrated. All calculations were done with the statistical software STATA 14.1.

## Results

### Phytochemical investigation of the plant species

**Fig 1:**
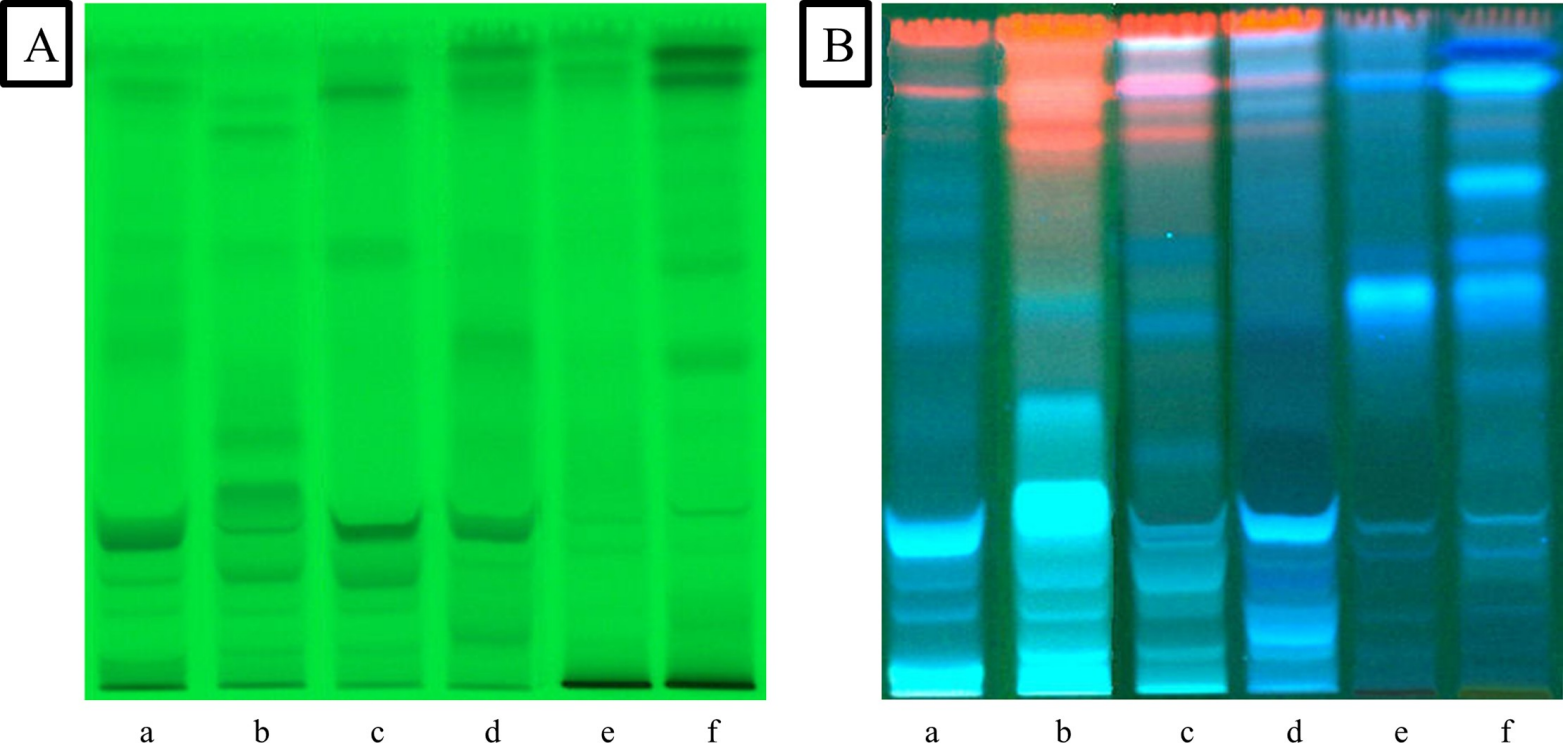
HPTLC chromatograms of (a) *R*. *officinalis*, (b) *S*. *sclarea*, (c) *O*. *vulgare*, (d) *T*. *longicaulis*, (e) *C*. *creticus* and (f) *C*. *monspeliensis* at 254 nm (**A**) and 366 nm (**B**) (mobile phase dichloromethane, methanol, water (70:30:4; v/v/v)).

**Fig 2:**
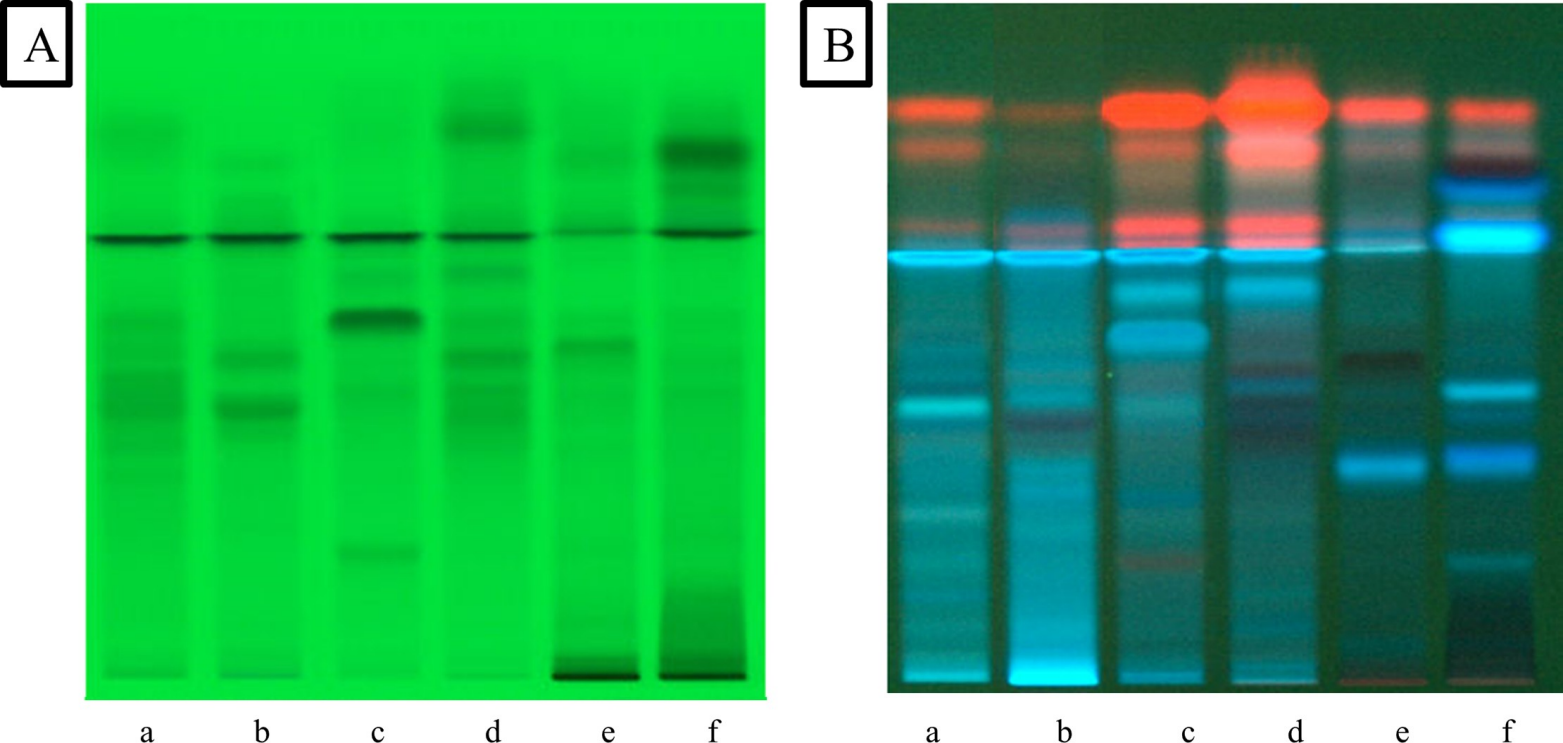
HPTLC chromatograms of (a) *R*. *officinalis*, (b) *S*. *sclarea*, (c) *O*. *vulgare*, (d) *T*. *longicaulis*, (e) *C*. *creticus* and (f) *C*. *monspeliensis* at 254 nm (**A**) and 366 nm (**B**) (mobile phase ethyl acetate, methanol, formic acid, water (50:10:7:1; v/v/v/v)).

The plants were extracted with methanol using the exhaustive and efficient ultrasound assisted extraction method. For a preliminary phytochemical fingerprinting of the extracts a method was developed with the aid of Camag HPTLC instrumentation. The HPTLC analysis showed that the methanol extracts of the aerial parts of the studied plants contained several bioactive compounds. Two different mobile phases were selected in order to get a better image of the compounds. Visualization of the plates was performed at 254 nm and 366 nm. The chromatograms (Figs 1A–1D and 2A–2D) showed spots, which are characteristic for several Lamiaceae species. In particular, phenolic acids and in particular rosmarinic acid appear as intense fluorescent blue spots in all four plant species (*O*. *vulgare*, *R*. *officinalis*, *T*. *longicaulis* and *S*. *sclarea*), while absorbance at 254 nm is typical of flavonoids. The two other plant species belong to the Cistaceae family (*C*. *creticus* ssp. *creticus* and *C*. *monspeliensis*). As shown in the HPTLC (Figs 1E, 1F, 2E and 2F), evident is the presence of tannins and some phenolic compounds.

**Fig 3:**
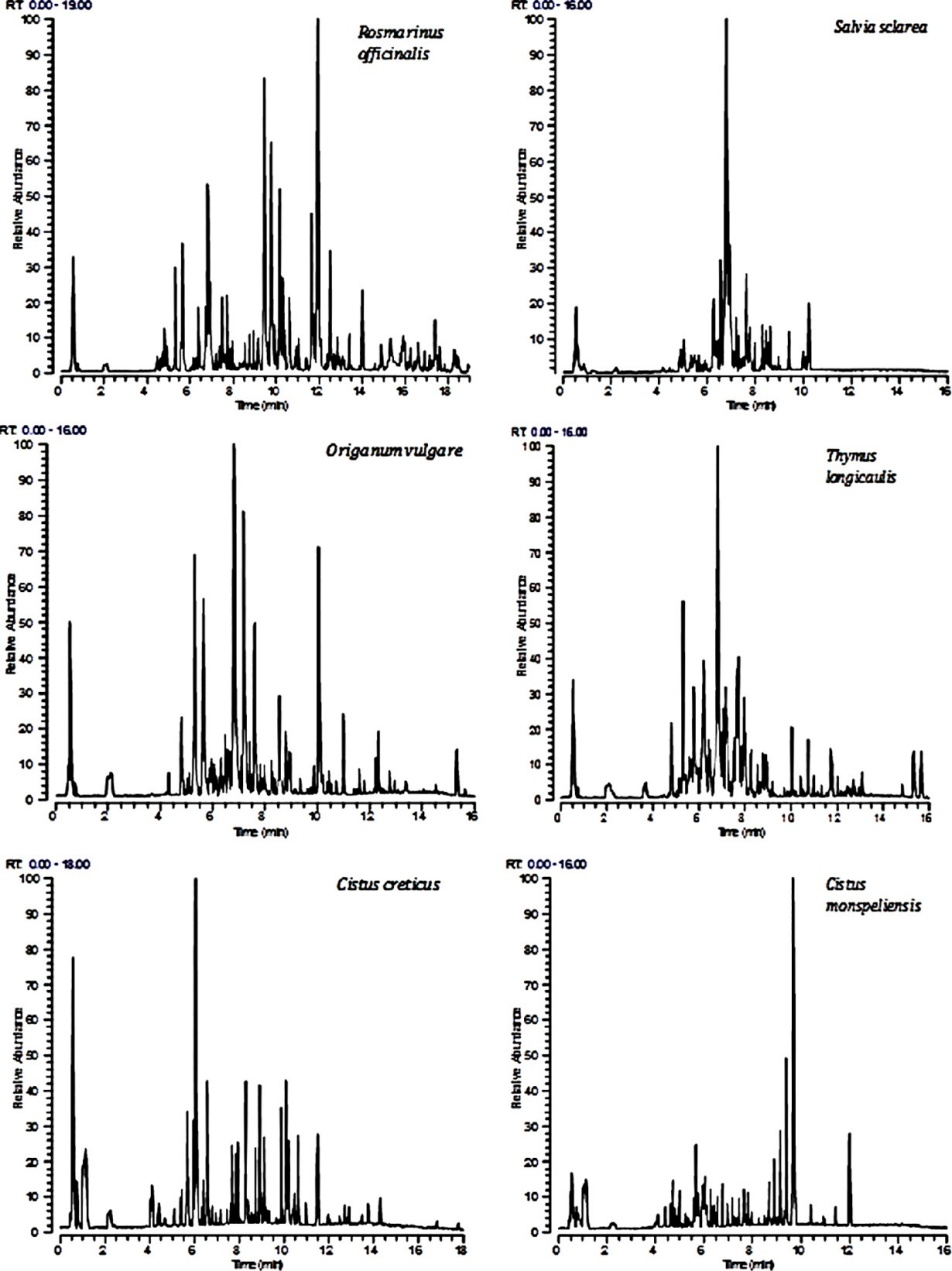
LC(ESI-)HRMS chromatograms of the methanol extracts of the tested plants.

Since the methanol extracts were particularly rich in metabolites of various classes of compounds a more comprehensive characterization of chemical composition of extracts was conducted by LC-HRMS. The methanol extracts were also analyzed by UPLC-ESI-HRMS in order to identify the active compounds of the extracts. Tables 1–6 summarize all the compounds characterized in the above extracts, including retention times (Rt), elemental composition of the detected ions (EC [M-H]-), experimental and theoretical m/z, Delta value (Δm, ppm), rings and double bonds equivalent (RDBeq), MS/MS fragments and identification of putative compounds. Metabolites were assigned by interpreting the mass spectra determined through their MS and MS/MS, taking into account all the data provided by literature [31–42]. Thus, a total of 183 compounds, among them a lot in common in all six extracts, belonging to various metabolite classes, were qualitatively characterized. Forty compounds were detected in *R*. *officinalis*, belonging to phenolic acids, flavonoids and triterpenic acids. 7-Methoxyrosmanol, rosmanol isomers and rosmarinic acid were the main metabolites in the methanol extract. Rosmarinic acid was also the major metabolite in the extract of *S*. *sclarea* while various flavonoids were also present. Flavonoids and phenolic acids were identified in the extract of *O*. *vulgare*. Rosmarinic acid was also in this extract the main metabolite along with salvianolic acids. *T*. *longicaulis* was rich in flavonoids and triterpenic acids, while in this extract rosmarinic acid was also the main metabolite. *Cistus* spp. were particularly rich in phenolic compounds and especially flavonoids. Quercetin 3-*O*-β-D-glucopyranoside was the major compound in *C*. *creticus*. In *C*. *monspeliensis* diterpenes were also detected in the methanol extract with cistodioic acid being the major compound. Fig 3 shows the base peak chromatograms (BPCs) of the analyzed extracts.

**Table 1:** LC(ESI-)HRMS of *R*. *officinalis* methanol extract.

No	Rt (min)	EC [M-H]^-^	Experimental	Theoretical	Δm ppm	RDBeq	Fragments MSMS *(m/z)*	Category of compound (*Identification)*
			[M-H]^-^ *m/z*					
**1**	0.55	C_7_H_11_O_6_	191.0563	191.05614	1.01	2.5	93, 127	***Quinic acid***
**2**	2.06	C_9_H_9_O_5_	197.0454	197.0455	-0.53	5.5	135, 179	***Syringic acid***
**3**	5.10	C_8_H_7_O_4_	167.0350	167.0350	0.49	5.5	146, 160	***Vanilic acid***
**4**	4.64	C_16_H_17_O_9_	353.0871	353.0878	-1.91	8.5	173, 179	***Chlorogenic acid (Isomer 1)***
**5**	4.90	C_16_H_17_O_9_	353.0871	353.0878	-1.91	8.5	nd	***Chlorogenic acid (Isomer 2)***
**6**	5.06	C_20_H_27_O_12_	459.1501	459.1508	-1.62	7.5	287, 196, 309, 167, 127	***Paeonolide***
**7**	5.63	C_12_H_17_O_4_	225.1132	225.1132	0.014	4.5	179	***12-Hydroxyjasmonic acid***
**8**	5.79	C_21_H_19_O_12_	463.0874	463.0882	-1.78	12.5	419	***6-Hydroxyluteolin-7-glucoside***
**9**	6.0	C_24_H_25_O_13_	521.1295	521.1301	-1.16	12.5	359	***Rosmarinic acid-3-D-glucoside***
**10**	6.10	C_27_H_29_O_15_	593.1506	593.1512	-0.94	13.5	473, 353, 503, 196	***Luteolin-7-O-rutinoside***
**11**	6.37	C_22_H_21_O_12_	477.1036	477.1038	-0.50	12.5	315, 197, 179,135	***Nepitrin***
**12**	6.65	C_22_H_19_O_10_	431.0977	431.09784	-1.44	12.5	269	***Apigenin-7-O-glucoside***
**13**	6.71	C_28_H_33_O_15_	609.1821	609.1825	-0.69	12.5	461	***Hesperidin***
**14**	6.78	C_18_H_15_O_8_	359.0772	359.0772	-0.1	11.5	161, 179, 197, 135	***Rosmarinic acid***
**15**	6.92	C_21_H_17_O_12_	461.0724	461.0725	-0.21	13.5	285	***Luteolin-3-glucuronide***
**16**	7.18	C_27_H_33_O_12_	549.1973	549.1977	-0.77	11.5	218, 475, 359	***Eucommin A***
**17**	7.23	C_29_H_25_O_14_	597.1244	597.1250	-0.98	17.5	555	***Pterogynoside***
**18**	7.43	C_32_H_29_O_15_	653.1501	653.1512	-1.60	18.5	503, 593, 331	***Feruloylnepitrin***
**19**	7.47	C_23_H_19_O_13_	503.0829	503.0831	-0.39	14.5	285, 399	***Luteolin-3′-O-(2″-O-acetyl)-β-D glucuronide (Isomer 1)***
**20**	7.61	C_15_H_9_O_6_	285.0402	285.0405	-0.94	11.5	146, 190, 262	***Luteolin***
**21**	7.73	C_23_H_19_O_13_	503.0827	503.0831	-0.75	14.5	285, 399	***Luteolin-3′-O-(2″-O-acetyl)-β-D glucuronide (Isomer 2)***
**22**	7.97	C_9_H_7_O_4_	179.0352	179.0350	1.14	6.5	135, 160	***Caffeic acid***
**23**	8.29	C_15_H_9_O_5_	269.0452	269.0455	-1.30	11.5	nd	***Apigenin***
**24**	8.43	C_16_H_11_O_6_	299.0558	299.05561	-0.98	11.5	179	***Diosmetin***
**25**	9.40	C_17_H_13_O_6_	313.0716	313.0718	-0.63	11.5	nd	***Cirsimaritin***
**26**	9.43	C_20_H_25_O_5_	345.1708	345.1707	0.08	8.5	301, 283	***Rosmanol (Isomer 1)***
**27**	9.79	C_20_H_25_O_5_	345.1706	345.1707	-0.35	8.5	283	***Rosmanol (Isomer 2)***
**28**	10.16	C_20_H_25_O_5_	345.1708	345.1707	0.08	8.5	nd	***Rosmanol (Isomer 3)***
**29**	11.79	C_19_H_23_O_3_	299.1651	299.1653	-0.66	8.5	284	***Miltipolone***
**30**	11.95	C_21_H_27_O_5_	359.1863	359.1864	-0.24	8.5	315, 287, 196, 272, 167	***7-Methoxyrosmanol***
**31**	12.52	C_20_H_23_O_5_	343.1549	343.1551	-0.46	9.5	325, 300, 287, 196, 315	***Rosmadial***
**32**	12.82	C_20_H_27_O_4_	331.1912	331.1915	-0.87	7.5	nd	***Carnosic acid***
**33**	12.84	C_20_H_27_O_3_	315.1963	315.1966	-0.78	7.5	287, 196, 167	***Rosmaridiphenol***
**34**	12.94	C_30_H_47_O_4_	471.3477	471.3480	-0.49	7.5	287, 196, 453, 287, 196, 167	***Anemosapogenin***
**35**	13.50	C_30_H_47_O_4_	471.3476	471.3480	-0.82	7.5	194, 116, 329	***Augustic acid***
**36**	14.01	C_21_H_29_O_4_	345.2068	345.2071	-0,91	7.5	301, 286	***Methyl carnosate***
**37**	15.28	C_30_H_47_O_3_	455.3527	455.3531	-0.82	7.5	452	***Betulinic acid***
**38**	15.32	C_30_H_47_O_3_	455.3524	455.3531	-1.43	7.5	407	***Oleanolic acid***
**39**	15.34	C_30_H_47_O_3_	455.3528	455.3531	-0.62	7.5	453	***Ursolic acid***
**40**	16.27	C_30_H_45_O_3_	453.3369	453.3374	-1.11	8.5	nd	***Micromeric acid***
**41**	18.23	C_30_H_47_O_4_	471.3474	471.3480	-0.49	8.5	467	***Benthamic acid***

**Table 2:** LC(ESI-)HRMS of *S*. *sclarea* methanol extract.

No	Rt (min)	EC [M-H]^-^	Experimental	Theoretical	Δm ppm	RDBeq	Fragments MSMS (m/z)	Category of compound (*Identification)*
			[M-H]^-^ *m/z*					
**1**	2.18	C_9_H_9_O_5_	197.0454	197.0455	-0.69	5.5	179, 153, 72	***Salvianic acid A***
**2**	4.86	C_9_H_7_O_4_	179.0350	179.0350	0.37	6.5	nd	***Caffeic acid***
**3**	5.34	C_27_H_29_O_15_	593.1501	593.1512	-1.76	13.5	287, 196, 323, 269, 194	***luteolin-7-O-rutinoside***
**4**	5.36	C_16_H_17_O_9_	353.0872	353.0878	-1.57	8.5	173, 179	***Chlorogenic acid***
**5**	5.78	C_15_H_9_O_7_	301.0349	301.0354	-1.43	11.5	nd	***Quercetin***
**6**	5.79	C_21_H_17_O_13_	477.0670	477.0675	-1.00	13.5	161, 301, 315, 359	***Quercetin-3-glucuronide (Miquelianin)***
**7**	5.80	C_21_H_17_O_13_	477.06670	477.06675	-1.00	13.5	315, 300, 287, 196	***6-hydroxyluteolin 7-O-glucuronide***
**8**	5.86	C_27_H_29_O_16_	609.1465	609.1461	0.71	13.5	301	***Rutin***
**9**	6.02	C_14_H_5_O_8_	300.9986	300.9990	-1.30	12.5	nd	***Ellagic acid***
**10**	6.16	C_21_H_20_O_12_	463.0876	463.0876	-1.32	12.5	419	***Hyperoside***
**11**	6.35	C_24_H_25_O_13_	521.1296	521.1301	-0.93	12.5	359	***Rosmarinic acid-3-D-glucoside***
**12**	6.66	C_7_H_6_O_3_	137.0248	137.0244	2.76	5.5	108, 93	***Hydroxybenzoic acid***
**13**	6.67	C_21_H_19_O_10_	431.0980	431.0984	-0.87	12.5	269	***Apigenin 7-O-D-glucoside***
**14**	6.72	C_21_H_17_O_11_	445.0775	445.0776	-0.40	13.5	287, 161, 196, 194, 115	***Apigenin-7-O-β-D glucuronide***
**15**	6.79	C_18_H_15_O_8_	359.0770	359.0772	-0.68	11.5	161, 179, 197	***Rosmarinic acid***
**16**	6.92	C_21_H_17_O_12_	461.0725	461.0725	-0.08	13.5	285	***Luteolin-7-O-β-D glucuronide***
**17**	7.11	C_27_H_21_O_12_	537.1035	537.1038	-0.62	17.5	135, 185, 25, 313, 461	***Salvianolic acid I***
**18**	7.18	C_27_H_21_O_12_	473.10855	473.10855	-0.81	13.5	287, 429, 196, 167	***Apigenin-acetylglucoside***
**19**	7.60	C_15_H_9_O_6_	285.0403	285.0405	-0.41	11.5	133, 151, 197, 213	***Luteolin***
**20**	7.76	C_16_H_11_O_7_	315.05063	315.05063	-1.26	11.5	83, 145, 187	***Rhamnetin***
**21**	7.80	C_16_H_11_O_6_	299.0558	299.0561	-1.08	11.5	285	***Diosmetin***
**22**	7.99	C_19_H_17_O_8_	373.0925	373.0929	-1.05	11.5	135, 175, 179, 197	***Methyl rosmarinate***
**23**	8.16	C_36_H_29_O_16_	717.1448	717.1461	-1.85	22.5	519	***Salvianolic acid B***
**24**	8.28	C_15_H_9_O_5_	269.0453	269.0455	-0.73	11.5	117, 151	***Apigenin***
**25**	9.32	C_16_H_11_O_6_	299.0557	299.0561	-1.39	11.5	299	***Hispidulin***
**26**	9.40	C_17_H_13_O_6_	313.0714	313.0718	-1.21	11.5	298, 196	***Cirsimaritin***
**27**	9.45	C_20_H_25_O_5_	345.1702	345.1707	-1.50	8.5	345	***Epi-rosmanol***
**28**	10.19	C_16_H_11_O_5_	283.0610	283.0612	-0.78	11.5	283	***Genkwanin***

**Table 3:** LC(ESI-)HRMS of *O*. *vulgare* methanol extract.

No	Rt (min)	EC [M-H]^-^	Experimental	Theoretical	Δm ppm	RDBeq	Fragments MSMS (m/z)	Category of compound (*Identification)*
			[M-H]^-^ *m/z*					
**1**	0.57	C_7_H_11_O_6_	191.0563	191.0561	1.01	2.5	127, 173, 71, 85	***Quinic acid***
**2**	2.09	C_9_H_9_O_5_	197.0454	197.0455	-0.69	5.5	179	***Salvianic acid A***
**3**	2.23	C_7_H_5_O_4_	153.0196	153.0193	1.50	5.5	nd	***Dihydroxybenzoic acid***
**4**	4.49	C_13_H_15_O_8_	299.0768	299.0772	-1.63	6.5	nd	***Hydroxybenzoic acid hexose***
**5**	4.86	C_9_H_7_O_4_	179.0350	179.0350	0.37	6.5	135, 167, 71	***Caffeic acid***
**6**	4.91	C_16_H_17_O_9_	353.0872	353.0878	-1.57	8.5	173, 179	***Chlorogenic acid***
**7**	5.08	C_8_H_7_O_4_	167.0352	167.0350	1.22	5.5	nd	***Vanilic acid***
**8**	5.11	C_27_H_29_O_16_	609.1453	609.1461	-1.38	13.5	489, 287, 196, 519, 167	***Luteolin-6*,*8-C-dihexose***
**9**	5.34	C_27_H_29_O_15_	593.1506	593.1512	-0.94	13.5	473, 353, 503, 196	***Apigenin-6*,*8-di-C-hexoside***
**10**	5.66	C_21_H_19_O_11_	447.0930	447.0933	-0.52	12.5	327, 353, 429, 287, 196	***Isoorientin***
**11**	5.67	C_9_H_7_O_3_	163.04033	163.04033	1.60	6.5	nd	***p-Coumaric acid***
**12**	6.08	C_21_H_19_O_10_	431.0978	431.0984	-1.30	12.5	341, 311, 287, 196	***Vitexin/Isovitexin***
**13**	6.14	C_15_H_11_O_7_	303.0508	303.0510	-0.60	10.5	287, 196, 96, 71	***Taxifolin***
**14**	6.21	C_11_H_9_O_7_	253.0352	253.0354	-0.80	7.5	nd	***Chrysin***
**15**	6.26	C_21_H_17_O_12_	461.0722	461.0725	-0.68	13.5	285	***Kaempferol-O glucuronide***
**16**	6.31	C_16_H_13_O_6_	301.0717	301.0718	-0.25	10.5	nd	***Hesperetin***
**17**	6.47	C_27_H_21_O_12_	537.1038	537.1038	-0.05	17.5	493	***Salvianolic acid I***
**18**	6.62	C_27_H_21_O_12_	537.1037	537.1038	-0.28	17.5	519	***Salvianolic acid H***
**19**	6.73	C_21_H_17_O_11_	445.0774	445.0776	-0.47	13.5	269, 287, 196, 302, 162, 124	***Apigenin 7-O glucuronide***
**20**	6.77	C_18_H_15_O_8_	359.0772	359.0772	-0.08	11.5	197, 161	***Rosmarinic acid***
**21**	6.89	C_27_H_21_O_12_	537.1038	537.1038	-0.05	17.5	493	***Lithospermic acid***
**22**	7.19	C_36_H_29_O_16_	717.1456	717.14561	-0.66	22.5	519, 475	***Salvianolic acid E/B***
**23**	7.38	C_26_H_19_O_10_	491.0981	491.0981	-0.58	17.5	311, 135	***Salvianolic acid C***
**24**	7.48	C_15_H_11_O_6_	287.0560	287.0561	-0.49	10.5	151, 135, 107	***Eriodictyol***
**25**	7.61	C_15_H_9_O_6_	285.0403	285.0405	-0.41	11.5	nd	***Kaempferol***
**26**	7.63	C_15_H_9_O_6_	285.0403	285.0405	-0.41	11.5	nd	***Luteolin***
**27**	7.94	C_28_H_31_O_15_	607.1664	607.1668	-0.65	13.5	299	***Diosmin***
**28**	8.04	C_17_H_13_O_7_	329.0663	329.0667	-1.00	11.5	314, 299	***Quercetin dimethyl ether***
**29**	8.28	C_15_H_9_O_5_	269.0455	269.0455	-0.16	11.5	nd	***Apigenin***
**30**	9.02	C_16_H_13_O_6_	301.2018	301.2020	-0.67	2.5	165, 135, 299	***Dihydro-kaempferide***

**Table 4:** LC(ESI-)HRMS of *T*. *longicaulis* methanol extract.

No	Rt (min)	EC [M-H]^-^	Experimental	Theoretical	Δm ppm	RDBeq	Fragments MSMS (m/z)	Category of compound *(Identification)*
			[M-H]^-^ *m/z*					
**1**	2.08	C_9_H_9_O_5_	197.04544	197.04544	-0.53	5.5	179	***3*,*4 Dihydroxyphenylacetic acid***
**2**	2.19	C_7_H_5_O_4_	153.0195	153.0193	1.20	5.5	109, 71	***Protocatechuic acid***
**3**	4.75	C_16_H_17_O_9_	353.0872	353.0878	-1.74	8.5	173, 179	***Chlorogenic acid***
**4**	4.86	C_9_H_7_O_4_	179.0350	179.0350	0.29	6.5	135, 71, 167	***Caffeic acid (Isomer I)***
**5**	5.13	C_9_H_7_O_4_	179.0350	179.0350	0.34	6.5	135, 71, 167	***Caffeic acid (Isomer II)***
**6**	5.33	C_27_H_29_O_15_	593.1506	593.1512	-0.94	13.5	473, 353, 196, 287, 503	***Apigenin diglucoside***
**7**	5.68	C_21_H_19_O_11_	447.09308	447.09308	-0.46	12.5	285	***Luteolin O-Hexoside***
**8**	5.75	C_12_H_19_O_12_	463.0879	463.0882	-0.60	12.5	301	***Quercetin-O-hexoside***
**9**	5.77	C_21_H_19_O_12_	463.0881	463.0882	-0.27	12.5	301, 151	***Quercetin 3-O-glucoside***
**10**	6.0	C_27_H_29_O_16_	609.1465	609.1461	0.71	13.5	301	***Rutin***
**11**	6.12	C_21_H_21_O_11_	449.1087	449.1089	-0.58	11.5	287, 196, 313	***Eriodictyol O-hexoside***
**12**	6.13	C_30_H_25_O_13_	593.1291	593.1303	-1.64	18.5	285, 196, 167, 478	***Kaempferol diglucoside***
**13**	6.20	C_21_H_19_O_11_	447.09308	447.09308	-0.46	12.5	285	***Luteolin 7-glucoside***
**14**	6.28	C_24_H_25_O_13_	521.1299	521.1301	-0.23	12.5	359, 287, 196	***Rosmarinic acid-3-D-glucoside***
**15**	6.81	C_18_H_15_O_8_	359.0769	359.0772	-0.85	11.5	161, 197, 179, 135	***Rosmarinic acid***
**16**	7.65	C_27_H_21_O_12_	537.1036	537.1038	-0.39	17.5	493	***Lithospermic acid A***
**17**	7.96	C_19_H_17_O_8_	373.0926	373.0929	-1.05	11.5	179	***Methyl rosmarinate***
**18**	8.28	C_15_H_9_O_5_	269.0454	269.0455	-0.50	11.5	nd	***Apigenin***
**19**	11.67	C_30_H_47_O_4_	471.3477	471.3480	-0.49	7.5	287	***Anemosapogenin***
**20**	12.07	C_30_H_47_O_4_	471.3476	471.3480	-0.82	7.5	287	***Augustic acid***
**21**	12.91	C_30_H_47_O_4_	471.3474	471.3480	-0.49	7.5	287	***Benthamic acid***
**22**	15.28	C_30_H_47_O_3_	455.3526	455.3531	-1.05	7.5	287	***Betulinic acid***
**23**	15.63	C_30_H_47_O_3_	455.3528	455.3531	-0.49	7.5	287	***Oleanolic acid***
**24**	15.65	C_30_H_47_O_3_	455.3530	455.3531	-0.15	7.5	287	***Ursolic acid***
**25**	16.29	C_30_H_45_O_3_	453.3369	453.3374	-1.11	8.5	287, 196	***Micromeric acid***

**Table 5:** LC(ESI-)HRMS of *C*. *creticus* methanol extract.

No	Rt (min)	EC [M-H]^-^	Experimental	Theoretical	Δm ppm	RDBeq	Fragments MSMS (m/z)	Category of compound (*Identification)*
			[M-H]^-^ *m/z*					
**1**	0.55	C_7_H_11_O_6_	191.0563	191.0561	1.01	2.5	173, 127, 85	***Quinic acid***
**2**	0.70	C_7_H_9_O_5_	173.0456	173.0455	0.18	3.5	nd	***Shikimic acid***
**3**	0.93	C_13_H_15_O_10_	331.0666	331.0671	-1.45	5.5	nd	***Glucogallin***
**4**	1.08	C_7_H_5_O_5_	169.0143	169.0142	0.57	5.5	125	***Gallic acid***
**5**	2.29	C_13_H_15_O_9_	315.0716	315.0722	-1.87	6.5	153, 287, 196	***Gentisoil glucoside***
**6**	2.38	C_15_H_13_O_7_	305.0661	305.0667	-1.78	9.5	179, 219, 165, 261	***Epigallocatechin***
**7**	4.04	C_12_H_13_O_8_	285.0613	285.0616	-1.09	6.5	153, 109	***Uralenneoside***
**8**	4.21	C_34_H_23_O_22_	783.0701	783.0686	1.81	23.5	nd	***Pedunculagin***
**9**	4.65	C_15_H_13_O_6_	289.07135	289.07135	-1.42	9.5	nd	***Cathechin***
**10**	4.67	C_20_H_19_O_14_	483.0714	483.0718	-1.52	9.5	nd	***Digaloil-β-D-glucopiranose***
**11**	4.80	C_27_H_21_O_18_	633.07220	633.07220	-1.78	17.5	300, 287, 196, 478	***Strictinin***
**12**	4.94	C_21_H_21_O_13_	481.0983	481.0988	-0.90	11.5	287, 196, 245	***Mirciaphenone B***
**13**	5.33	C_27_H_29_O_15_	593.1506	593.1512	-0.94	13.5	473	***Apigenin diglucoside***
**14**	5.64	C_27_H_29_O_17_	625.1401	625.1410	-1.51	13.5	463, 301	***Quercetin diglucoside***
**15**	5.65	C_21_H_19_O_13_	479.08261	479.08261	-1.04	12.5	nd	***Myricetin-3-O-glucoside***
**16**	5.82	C_19_H_13_O_12_	433.0406	433.04012	-1.38	13.5	301	***Ellagic acid-7-xyloside***
**17**	6.01	C_27_H_29_O_16_	609.1450	609.1461	-1.78	13.5	287, 301, 196, 478	***Kaempferol diglucoside***
**18**	6.06	C_27_H_29_O_16_	609.1446	609.1461	-2.48	13.5	301	***Rutin***
**19**	6.02	C_21_H_19_O_12_	463.0881	463.0882	-0.27	12.5	301, 151	***Quercetin 3-O-β-D-glucopyranoside***
**20**	6.15	C_21_H_19_O_12_	463.0877	463.0882	-1.06	12.5	316	***Myricetin-3-O-rhamnoside***
**21**	6.34	C_14_H_5_O_8_	300.9988	300.9990	-0.59	12.5	nd	***Ellagic acid***
**22**	6.37	C_27_H_29_O_15_	593.1506	593.1512	-0.94	13.5	287	***Luteolin-7-O-rutinoside***
**23**	6.56	C_21_H_19_O_11_	447.0930	447.0933	-0.73	12.5	301	***Isoorientin***
**24**	7.0	C_30_H_25_O_16_	625.1188	625.1199	-1.78	18.5	316, 479	***Myricetin-O-rhamnoside-O-hexoside***
**25**	7.65	C_30_H_25_O_13_	593.1294	593.1301	-1.28	18.5	285, 447	***Kaempferol -O-rhamnoside-O-hexoside***
**26**	7.81	C_30_H_25_O_13_	593.1291	593.1303	-1.64	18.5	285, 447	***Kaempferol diglucoside***
**27**	8.27	C_15_H_9_O_5_	269.0454	269.0455	-0.39	11.5	nd	***Apigenin***
**28**	8.72	C_16_H_11_O_6_	299.05585	299.05585	-0.88	11.5	284, 196	***Kaempferol methylether***
**29**	10.08	C_16_H_11_O_5_	283.0611	283.0612	-0.35	11.5	268	***Apigenin methylether***
**30**	10.09	C_16_H_11_O_5_	283.0608	283.0612	-1.32	11.5	283	***Apigenin methylether***

**Table 6:** LC(ESI-)HRMS of *C*. *monspeliensis* methanol extract.

No	Rt (min)	EC [M-H]^-^	Experimental	Theoretical	Δm ppm	RDBeq	Fragments MSMS (m/z)	Category of compound (*Identification)*
			[M-H]^-^ *m/z*					
**1**	0.56	C_7_H_11_O_6_	191.0562	191.0561	0.61	2.5	173, 127, 85	***Quinic acid***
**2**	0.69	C_7_H_9_O_5_	173.0456	173.0455	0.18	3.5	nd	***Shikimic acid***
**3**	1.15	C_13_H_15_O_10_	331.0667	331.0671	0.57	5.5	nd	***Glucogallin***
**4**	1.08	C_7_H_5_O_5_	169.0143	169.0142	0.57	5.5	125	***Gallic acid***
**5**	2.19	C_7_H_5_O_4_	153.0195	153.0193	1.50	5.5	109, 71	***Dihydroxybenzoic acid***
**6**	2.29	C_13_H_15_O_9_	315.0716	315.0722	-1.87	6.5	153, 287, 196	***Gentisoil glucoside***
**7**	4.06	C_12_H_13_O_8_	285.0611	285.0616	-1.84	6.5	153, 109	***Uralenneoside***
**8**	4.40	C_8_H_7_O_5_	183.0299	183.0299	-0.05	5.5	nd	***Methyl gallate***
**9**	4.64	C_27_H_29_O_17_	625.1401	625.1410	-1.51	13.5	463, 301	***Quercetin diglucoside***
**10**	5.35	C_27_H_29_O_15_	593.1506	593.1512	-0.94	13.5	287	***Luteolin-7-O-rutinoside***
**11**	5.65	C_21_H_19_O_13_	479.08261	479.08261	-1.04	12.5	316, 287, 196	***Myricetin-3-O-glucoside***
**12**	5.80	C_19_H_13_O_12_	433.0406	433.04012	-1.45	13.5	301	***Ellagic acid-7-xyloside***
**13**	5.95	C_20_H_17_O_12_	449.0721	449.0725	-0.97	12.5	317, 287, 196	***Myricetin 3'-xyloside***
**14**	6.00	C_14_H_5_O_8_	300.9988	300.9990	-0.59	12.5	nd	***Ellagic acid***
**15**	6.08	C_21_H_19_O_12_	463.0881	463.0882	-0.27	12.5	301, 151	***Quercetin 3-O-β-D-glucopyranoside***
**16**	6.15	C_21_H_19_O_12_	463.0877	463.0882	-1.06	12.5	316	***Myricetin-3-O-rhamnoside***
**17**	6.34	C_20_H_17_O_11_	433.0775	433.0776	-0.41	12.5	301, 287, 196, 167, 126	***Quercetin 3-alpha-L-arabinofuranoside (Avicularin Isomer 1)***
**18**	6.47	C_20_H_17_O_11_	433.0775	433.0776	-0.41	12.5	301, 287, 196, 167, 126	***Quercetin 3-alpha-L-arabinofuranoside (Avicularin Isomer 2)***
**19**	6.55	C_21_H_19_O_11_	447.0928	447.0933	-1.00	12.5	301	***Isoorientin***
**20**	6.74	C_8_H_7_O_5_	293.1390	293.1394	-1.49	6.5	249, 274, 287, 205, 196	***Propanoic acid methyl ester***
**21**	6.95	C_30_H_25_O_16_	625.1188	625.1199	-1.78	18.5	316, 479	***Myricetin-O-rhamnoside-O-hexoside***
**22**	7.61	C_30_H_25_O_13_	593.1294	593.1301	-1.28	18.5	285, 447	***Kaempferol-O-rhamnoside-O-hexoside***
**23**	7.69	C_15_H_9_O_7_	301.0349	301.0354	-1.43	11.5	178, 151, 196, 287, 71	***Quercetin***
**24**	7.79	C_30_H_25_O_13_	593.1291	593.1303	-1.64	18.5	285, 447	***Kaempferol diglucoside***
**25**	8.04	C_17_H_13_O_7_	329.0663	329.0667	-1.00	11.5	314, 287, 196, 167	***Quercetin dimethyl ether***
**26**	9.14	C_18_H_15_O_8_	359.0772	359.0772	-0.08	11.5	344, 329	***Rosmarinic acid***
**27**	9.39	C_20_H_29_O_5_	349.2019	349.2020	-0.32	6.5	305	***Andrographolide***
**28**	9.66	C_20_H_31_O_4_	335.2228	335.2228	0.16	5.5	291, 287, 196, 126, 100	***Cistodioic acid***
**29**	10.95	C_19_H_17_O_8_	373.0923	373.0929	-1.70	11.5	358, 287, 196	***Eriostemin***
**30**	12.0	C_22_H_33_O_5_	377.2330	377.2333	-0.91	6.5	nd	***Laurifolic acid***

### *C*. *creticus* and *C*. *monspeliensis* exhibited high antibacterial and antibiofilm activity against obligate anaerobes and *S*. *mutans*, respectively

**Fig 4:**
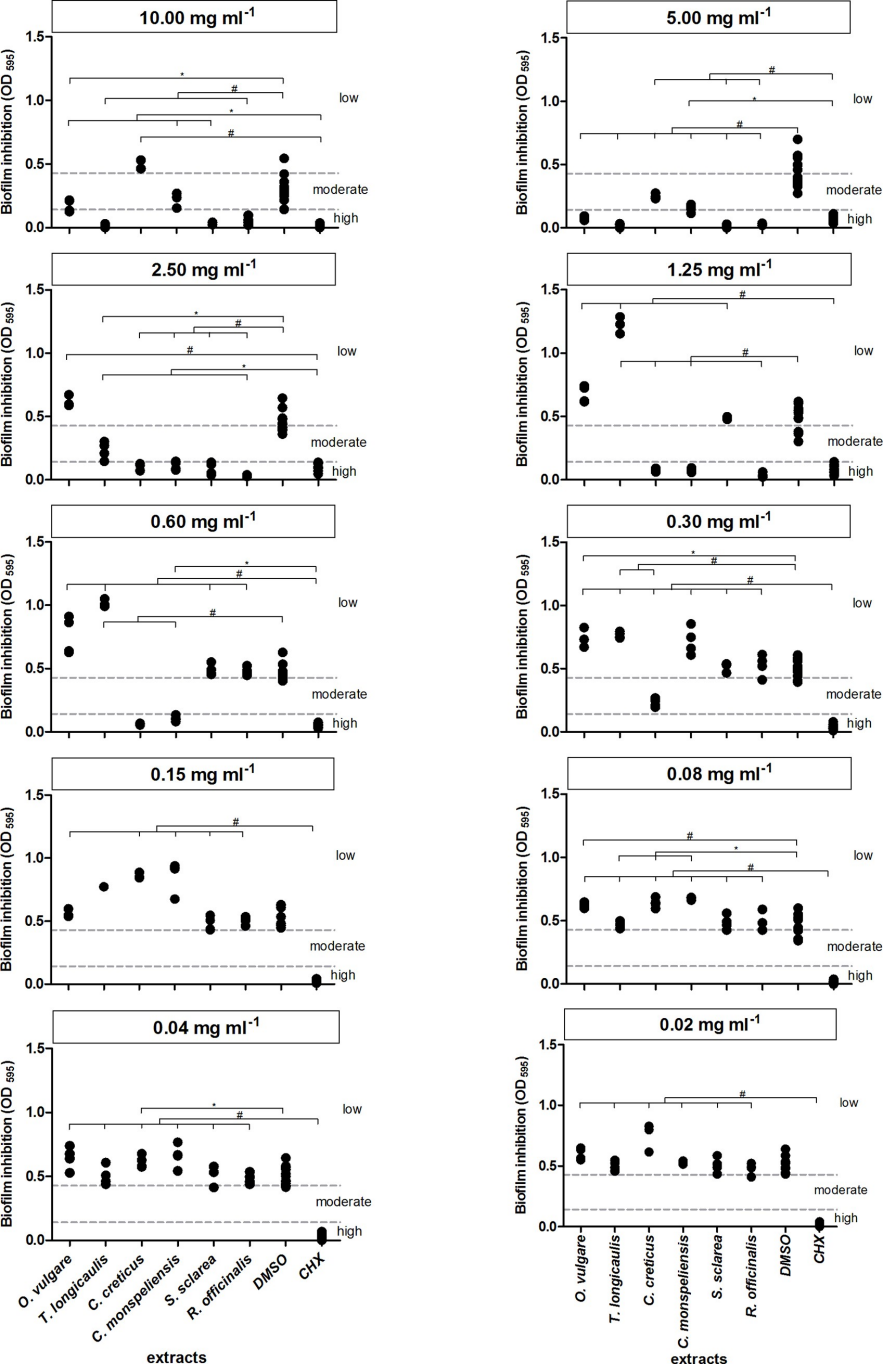
Diagrams depicting the antibiofilm effect of six Mediterranean herb extracts against *S*. *mutans*. The low and high cut-off OD_595_ values were estimated at 0.143 and 0.428, respectively. High *S*. *mutans* biofilm inhibition is exhibited at OD_595_ values ≤0.143, whereas *S*. *mutans* moderate biofilm formation is displayed at 0.143 ≤ OD_595_ values ≤0.428. DMSO and CHX concentrations are shown for each extract concentration. Hash symbols and asterisks represent statistically significant differences (p < 0.01 and p < 0.05), respectively.

The mean MIC / MBC values for the methanol extract of *C*. *creticus* are shown in Table 7. Under its influence, especially the growth of the obligate anaerobes was efficiently reduced at low inhibitory concentrations ranging from 0.08 mg ml^-1^ (*P*. *gingivalis*, *P*. *micra*) to 0.60 mg ml^-1^ (*F*. *nucleatum*), while MBC values varied from 0.15 mg ml^-1^ (*P*. *gingivalis*, *P*. *micra*) to 0.60 mg ml^-1^ (*P*. *intermedia*, *F*. *nucleatum*). Likewise, *S*. *oralis* was eliminated at a bactericidal *C*. *creticus* concentration of 0.60 mg ml^-1^. Additionally, the extract was very effective against *S*. *aureus* (MIC = 0.60 mg ml^-1^), whereas it had a moderate effect on *S*. *sobrinus* and *E*. *faecalis* with a MIC value of 5.00 mg ml^-1^. No significant antimicrobial effect was observed on *S*. *mutans*, *C*. *albicans* and *E*. *coli*. The biofilm plate assay revealed a moderate reduction of biofilm production as shown in Fig 4. The biofilm formation of *S*. *mutans* was significantly inhibited (mean OD_595_ = 0.231) at a *C*. *creticus* concentration of 0.60 mg ml^-1^ (low cut-off value OD_595_ = 0.143), while high *S*. *mutans* biofilm formation was observed at 0.15 mg ml^-1^ (high cut-off value OD_595_ = 0.428).

**Table 7:** Antimicrobial activity in mg ml^-1^ of *C*. *creticus* methanol extract.

*C*. *creticus*				
Sample	Methanol extract DMSO (%)			
(in mg ml^-1^)	MIC	MBC	MIC	MBC
*Streptococcus mutans* DSM 20523	5.00	10.00	5.00	NA
*Streptococcus sobrinus* DSM 20381	5.00	10.00	20.00	20.00
*Streptococcus oralis* ATCC 35037	0.30	0.60	10.00	20.00
*Enterococcus faecalis* ATCC 29212	5.00	10.00	20.00	NA
*Candida albicans* DSM 1386	10.00	10.00	10.00	20.00
*Escherichia coli* ATCC 25922	NA	NA	20.00	NA
*Staphylococcus aureus* ATCC 25923	0.60	2.50	20.00	NA
*Porphyromonas gingivalis* W381	0.08	0.15	20.00	20.00
*Prevotella intermedia* MSP 34	0.30	0.60	5.00	5.00
*Fusobacterium nucleatum* ATCC 25586	0.60	0.60	10.00	10.00
*Parvimonas micra* ATCC 23195	0.08	0.15	10.00	20.00

NA: No activity observed, MIC or MBC of extracts and DMSO at 10.00 mg ml^-1^ and 20%, respectively.

MIC = extract concentration at which the OD measurement revealed minimal bacterial growth.

MBC = extract concentration at which a 3-Log reduction (99.9%) of the bacterial growth was induced.

Table 8 summarizes the MIC and MBC values for the methanol extract of *C*. *monspeliensis*. In general, *C*. *monspeliensis* exhibited significant bactericidal effects against the obligate anaerobes with a MIC range from 0.04 mg ml^-1^ (*P*. *gingivalis*, *P*. *micra*) to 0.60 mg ml^-1^ (*F*. *nucleatum*), while the extract had no antimicrobial impact on *E*. *coli* and *C*. *albicans*. A moderate inhibitory effect was observed for *S*. *mutans* (MIC = 2.50 mg ml^-1^). The MIC and MBC values were estimated at 0.15 mg ml^-1^ and 0.60 mg ml^-1^ for *S*. *oralis*, whereas *E*. *faecalis* was less susceptible to *C*. *monspeliensis* (MIC = 5.00 mg ml^-1^; MBC = 10.00 mg ml^-1^). The methanol extract of *C*. *monspeliensis* had a high inhibitory effect on *S*. *mutans* biofilm formation (Fig 4) at a concentration of 0.60 mg ml^-1^ (mean OD_595_ = 0.106).

**Table 8:** Antimicrobial activity in mg ml^-1^ of *C*. *monspeliensis* methanol extract.

*C*. *monspeliensis*				
Sample	Methanol extract DMSO (%)			
(in mg ml^-1^)	MIC	MBC	MIC	MBC
*Streptococcus mutans* DSM 20523	2.50	NA	10.00	NA
*Streptococcus sobrinus* DSM 20381	2.50	5.00	20.00	20.00
*Streptococcus oralis* ATCC 35037	0.15	0.60	10.00	20.00
*Enterococcus faecalis* ATCC 29212	5.00	10.00	20.00	NA
*Candida albicans* DSM 1386	5.00	10.00	5.00	NA
*Escherichia coli* ATCC 25922	NA	NA	20.00	NA
*Staphylococcus aureus* ATCC 25923	0.60	2.50	20.00	NA
*Porphyromonas gingivalis* W381	0.04	0.15	20.00	20.00
*Prevotella intermedia* MSP 34	0.08	0.08	2.50	5.00
*Fusobacterium nucleatum* ATCC 25586	0.60	0.60	10.00	10.00
*Parvimonas micra* ATCC 23195	0.04	0.08	10.00	20.00

NA: No activity observed, MIC or MBC of extracts and DMSO at 10.00 mg ml^-1^ and 20%, respectively.

MIC = extract concentration at which the OD measurement revealed minimal bacterial growth.

MBC = extract concentration at which a 3-Log reduction (99.9%) of the bacterial growth was induced.

### *R*. *officinalis* and *S*. *sclarea* significantly reduced growth of all oral bacteria and biofilm growth of *S*. *mutans*

In general, the methanol extract of *R*. *officinalis* was extremely effective against all tested pathogens (Table 9). The MIC values ranged from 0.08 mg ml^-1^ (*P*. *micra*) to 5.00 mg ml^-1^ (*E*. *faecalis*), with the highest efficacy among the streptococci on *S*. *oralis* at 0.30 mg ml^-1^. Similarly, the MBC values showed bactericidal effects in a range from 0.15 mg ml^-1^ (*P*. *micra*) to 5.00 mg ml^-1^ (*E*. *faecalis*). The extract had no significant effects on *C*. *albicans* and *E*. *coli*. The biofilm formation of *S*. *mutans* substantially decreased (mean OD_595_ = 0.038) at a *R*. *officinalis* concentration of 1.25 mg ml^-1^ (Fig 4).

**Table 9:** Antimicrobial activity in mg ml^-1^ of *R*. *officinalis* methanol extract.

*R*. *officinalis*				
Sample	Methanol extract DMSO (%)			
(in mg ml^-1^)	MIC	MBC	MIC	MBC
*Streptococcus mutans* DSM 20523	0.60	2.50	5.00	NA
*Streptococcus sobrinus* DSM 20381	1.25	2.50	20.00	NA
*Streptococcus oralis* ATCC 35037	0.30	0.60	20.00	20.00
*Enterococcus faecalis* ATCC 29212	5.00	5.00	20.00	NA
*Candida albicans* DSM 1386	10.00	10.00	10.00	NA
*Escherichia coli* ATCC 25922	10.00	10.00	20.00	NA
*Staphylococcus aureus* ATCC 25923	0.60	1.25	20.00	NA
*Porphyromonas gingivalis* W381	0.15	0.30	20.00	20.00
*Prevotella intermedia* MSP 34	0.30	0.60	5.00	5.00
*Fusobacterium nucleatum* ATCC 25586	1.25	1.25	10.00	20.00
*Parvimonas micra* ATCC 23195	0.08	0.15	5.00	20.00

NA: No activity observed, MIC or MBC of extracts and DMSO at 10.00 mg ml^-1^ and 20%, respectively.

MIC = extract concentration at which the OD measurement revealed minimal bacterial growth.

MBC = extract concentration at which a 3-Log reduction (99.9%) of the bacterial growth was induced.

The mean MIC and MBC values for the methanol extract of S. *sclarea* are presented in Table 10. The MIC values vary between 0.08 mg ml^-1^ (*P*. *micra*) and 2.50 mg ml^-1^ (*S*. *sobrinus*). *S*. *sclarea* extract at 0.15 mg ml^-1^ induced a 3-Log reduction of *P*. *gingivalis* and *P*. *micra*, whereas a higher *S*. *sclarea* concentration at 5.00 mg ml^-1^ was needed to eradicate *S*. *mutans* and *S*. *sobrinus*. *E*. *coli*, *E*. *faecalis* and *C*. *albicans* were not affected by the extract. Significant inhibition of biofilm formation of *S*. *mutans* (mean OD_595_ = 0.088) by *S*. *sclarea* was shown at a concentration of 2.50 mg ml^-1^ (Fig 4).

**Table 10:** Antimicrobial activity in mg ml^-1^ of *S*. *sclarea* methanol extract.

*S*. *sclarea*				
Sample	Methanol extract DMSO (%)			
(in mg ml^-1^)	MIC	MBC	MIC	MBC
*Streptococcus mutans* DSM 20523	1.25	5.00	5.00	NA
*Streptococcus sobrinus* DSM 20381	2.50	5.00	20.00	NA
*Streptococcus oralis* ATCC 35037	0.60	0.60	20.00	20.00
*Enterococcus faecalis* ATCC 29212	10.00	10.00	20.00	NA
*Candida albicans* DSM 1386	10.00	10.00	10.00	NA
*Escherichia coli* ATCC 25922	10.00	10.00	20.00	NA
*Staphylococcus aureus* ATCC 25923	1.25	1.25	20.00	NA
*Porphyromonas gingivalis* W381	0.15	0.15	20.00	20.00
*Prevotella intermedia* MSP 34	0.15	0.30	5.00	5.00
*Fusobacterium nucleatum* ATCC 25586	0.60	0.60	10.00	20.00
*Parvimonas micra* ATCC 23195	0.08	0.15	5.00	20.00

NA: No activity observed, MIC or MBC of extracts and DMSO at 10.00 mg ml^-1^ and 20%, respectively.

MIC = extract concentration at which the OD measurement revealed minimal bacterial growth.

MBC = extract concentration at which a 3-Log reduction (99.9%) of the bacterial growth was induced.

### *O*. *vulgare* and *T*. *longicaulis* demonstrated significant bactericidal activity against obligate anaerobes but low antibiofilm activity against *S*. *mutans*

The methanol extract of *O*. *vulgare* eradicated the tested oral bacteria in a range of MBC values from 0.30 mg ml^-1^ (*P*. *micra*) to 5.00 mg ml^-1^ for *E*. *faecalis*, *S*. *mutans* and *S*. *sobrinus*, as shown in Table 11. The extract had nearly no antimicrobial impact on *C*. *albicans*, *E*. *coli* and the obligate anaerobe *P*. *intermedia*. The most significant inhibitory effect was induced at a *O*. *vulgare* concentration of 0.30 mg ml^-1^ on *P*. *gingivalis* and *P*. *micra* and at 0.60 mg ml^-1^ on *S*. *oralis*, respectively. Biofilm formation of *S*. *mutans* was significantly reduced (mean OD_595_ = 0.081) at 5.00 mg ml^-1^ *O*. *vulgare* extract, whereas lower extract concentration had no impact on *S*. *mutans* biofilms (Fig 4).

**Table 11:** Antimicrobial activity in mg ml^-1^ of *O*. *vulgare* methanol extract.

*O*. *vulgare*				
Sample	Methanol extract DMSO (%)			
(in mg ml^-1^)	MIC	MBC	MIC	MBC
*Streptococcus mutans* DSM 20523	2.50	5.00	5.00	NA
*Streptococcus sobrinus* DSM 20381	2.50	5.00	20.00	NA
*Streptococcus oralis* ATCC 35037	0.60	1.25	10.00	20.00
*Enterococcus faecalis* ATCC 29212	5.00	5.00	20.00	NA
*Candida albicans* DSM 1386	10.00	10.00	10.00	10.00
*Escherichia coli* ATCC 25922	10.00	10.00	20.00	NA
*Staphylococcus aureus* ATCC 25923	1.25	1.25	20.00	NA
*Porphyromonas gingivalis* W381	0.30	0.60	20.00	20.00
*Prevotella intermedia* MSP 34	2.50	2.50	5.00	5.00
*Fusobacterium nucleatum* ATCC 25586	1.25	1.25	10.00	10.00
*Parvimonas micra* ATCC 23195	0.30	0.30	10.00	20.00

NA: No activity observed, MIC or MBC of extracts and DMSO at 10.00 mg ml^-1^ and 20%, respectively.

MIC = extract concentration at which the OD measurement revealed minimal bacterial growth.

MBC = extract concentration at which a 3-Log reduction (99.9%) of the bacterial growth was induced.

The antimicrobial activity of *T*. *longicaulis* on the tested bacterial strains and *C*. *albicans* is displayed in Table 12. The MIC values varied from 0.08 mg ml^-1^ (*P*. *micra*) to 2.50 mg ml^-1^ (*S*. *sobrinus*, *E*. *coli*). The MBC values demonstrated the persistence of *C*. *albicans* and *E*. *coli* in the presence of 10.00 mg ml^-1^ *T*. *longicaulis* methanol extract, whereas all other strains were killed from 0.15 mg ml^-1^ (*P*. *micra*) to 2.50 mg ml^-1^ (*S*. *sobrinus*, *E*. *faecalis*). *T*. *longicaulis* inhibited strongly biofilm formation by *S*. *mutans* at 5.00 mg ml^-1^ (mean OD_595_ = 0.013) but also no statistically significant difference to CHX was detected at 2.50 mg ml^-1^ (Fig 4).

**Table 12:** Antimicrobial activity in mg ml^-1^ of *T*. *longicaulis* methanol extract.

*T*. *longicaulis*				
Sample	Methanol extract DMSO (%)			
(in mg ml^-1^)	MIC	MBC	MIC	MBC
*Streptococcus mutans* DSM 20523	0.60	1.25	5.00	NA
*Streptococcus sobrinus* DSM 20381	2.50	2.50	10.00	NA
*Streptococcus oralis* ATCC 35037	0.60	1.25	20.00	20.00
*Enterococcus faecalis* ATCC 29212	1.25	2.50	20.00	NA
*Candida albicans* DSM 1386	5.00	10.00	10.00	NA
*Escherichia coli* ATCC 25922	2.50	10.00	20.00	NA
*Staphylococcus aureus* ATCC 25923	0.60	0.60	20.00	NA
*Porphyromonas gingivalis* W381	0.15	0.30	20.00	20.00
*Prevotella intermedia* MSP 34	0.30	0.60	2.50	2.50
*Fusobacterium nucleatum* ATCC 25586	0.60	0.60	5.00	10.00
*Parvimonas micra* ATCC 23195	0.08	0.15	5.00	10.00

NA: No activity observed, MIC or MBC of extracts and DMSO at 10.00 mg ml^-1^ and 20%, respectively.

MIC = extract concentration at which the OD measurement revealed minimal bacterial growth.

MBC = extract concentration at which a 3-Log reduction (99.9%) of the bacterial growth was induced.

## Discussion

In this report all tested extracts effectively inhibited mainly the anaerobic oral microorganisms and in concentrations ≥0.3 mg ml^-1^ had moderate to high antibiofilm activity against *S*. *mutans* comparable to that of CHX. Lamiaceae is a family of great diversity and variety, containing important medicinal herbs with diverse biological properties. Most of the plants are aromatic and possess essential oils; however, they also contain important phenolic compounds. Additionally, *Cistus* species are widely spread in Europe and some, such as *C*. *monspeliensis* and *C*. *creticus* are typical components of the Mediterranean flora. Preliminary phytochemical analysis was performed with HPTLC, which is most recent evolution of planar chromatography, specifically tailored for the analysis of natural products. The microtiter plate test is an accepted standard biofilm assay, which has been used frequently in literature due to its practicability. It has the advantages to allow testing of different bacterial species or substances.

To date, most of the studies found in literature [43–45] have mainly reported on the antimicrobial effects of essential oils from *C*. *creticus*, *C*. *monspeliensis*, *O*. *vulgare*, *R*. *officinalis*, *S*. *sclarea* and *T*. *longicaulis* on “non-oral” bacterial pathogens and *C*. *albicans*. To the best of our knowledge, this is the first report on the antibacterial and antibiofilm effectiveness of methanol extracts from the aforementioned herbs against various oral pathogens.

In the present report, *C*. *creticus* and *C*. *monspeliensis* extracts inhibited effectively obligate anaerobes such as *P*. *gingivalis*, *P*. *micra* and *P*. *intermedia*. To date, several studies [43, 45–48] have confirmed the enhanced antimicrobial activity of *Cistus* spp. against diverse “non-oral” bacteria and fungi. Interestingly, six out of seven labdane-type isolated diterpenes from leaves of *C*. *creticus* L. showed inhibitory effects against the Gram-negative *Pseudomonas aeruginosa* and *Klebsiella pneumoniae* [43]. As the methanol extract of *C*. *creticus* exhibited no significant antimicrobial activity against *E*. *coli* in this study, the resulted MIC value was comparable with the relative high MIC ≥3 mg ml^-1^ of a hexane leaf extract of *C*. *creticus* L. [47]. In another report, Bouamama *et al*. [45] tested *C*. *monspeliensis* methanol extract against *E*. *coli* and exhibited no activity of the extract accounting for a MIC of 25 mg ml^-1^.

In general, facultative anaerobic Gram-negative bacteria are more resistant to the *Cistus* spp. extracts than Gram-positive microorganisms [48], while obligate anaerobes are more sensitive to *Cistus* spp. treatment. This is due to the fact that the outer bacterial cell membrane of Gram-negative bacteria serves as a dense permeability barrier that hinders the penetration of lipophilic molecules [49, 50]. The Gram-positive bacteria with thicker cell walls than the Gram-negative bacteria also develop defense strategies against antimicrobials such as the production of extracellular proteases and the chemical modification of cell membrane or cell wall [51, 52]. In an earlier *in situ* report [53], rinsing of the oral cavity with Cistus tea resulted in the reduction of adherent bacteria on enamel surfaces. In the present report, the high antibacterial activity of the *Cistus* spp. extracts against the Gram-positive *S*. *oralis* and *S*. *aureus* contradicted the significantly lower susceptibility of *S*. *aureus* to *C*. *monspeliensis* methanol extract (MIC = 25 mg ml^-1^) demonstrated by Bouamama *et al*. [45]. Nevertheless, the hexane leaf extract of *C*. *creticus* showed high bacterial growth inhibition of *S*. *aureus* and *E*. *faecalis* (MIC = 0.5 mg ml^-1^) in an earlier report by Anastasaki *et al*. [47]. The conflicting outcomes can be ascribed to the differing extraction methods used. In regard with the antifungal activity, even after the application of high-concentrated extracts, *C*. *albicans* could not be effectively killed [43, 45–47].

The tested rosemary and sage extracts significantly reduced the growth of all screened oral bacteria such as streptococci and *E*. *faecalis*. Although the methanol extract of *R*. *officinalis* reduced *S*. *mutans* (MIC = 0.60 mg ml^-1^) effectively in this report, the inhibition level of *S*. *sobrinus* and *F*. *nucleatum* (MIC = 1.25 mg ml^-1^) was moderate. Surprisingly, the phenols carnosic acid and carnosol enabled the activity of various conventional antibiotics against overexpressing efflux pumps in *S*. *aureus* strains [54]. In contrast, an rosemary EO tested by Hammer *et al*. [55] failed to inhibit *E*. *faecalis* at 2% (v/v).

In a randomized double-blind placebo-controlled trial a polyherbal mouthwash rinsing containing *R*. *officinalis* among three hydroalcoholic extracts, showed a high antibacterial efficacy comparable to 0.2% (w/v) CHX in the treatment of gingivitis [56]. Since new rosemary compounds have been isolated lately [57], further antimicrobial screening of rosemary is required.

The positive antimicrobial effects of *S*. *sclarea* methanol extract are in agreement with the MIC values against *S*. *aureus* and *E*. *coli* revealed by Firuzi *et al*. [58], but do not correspond to the low antibacterial activity against *S*. *aureus* shown by Stagos *et al*. [59]. The results are in accordance to the low susceptibility shown by Hammer *et al*. [55]. In the present study, the high antibacterial activity against facultative anaerobic oral pathogens was confirmed [60]. Finally, the main *S*. *sclarea* components, the diterpenoids salvipisone and aethiopinone, revealed both bactericidal and antibiofilm activity against *S*. *aureus* and *S*. *epidermidis* strains [61, 62].

Although the *O*. *vulgare* methanol extract effectively killed Gram-negative obligate anaerobes in this study, *S*. *aureus* and *E*. *coli* were not as susceptible to extract treatment contrary to the outcomes of previous reports on an ethanol extract [63] and an EO [55]. The discrepancies can be attributed to different extraction solvents used, MIC method or different chemical composition of the extract used.

In the present report, *T*. *longicaulis* eradicated both Gram-positive and Gram-negative bacteria confirming the results of earlier studies, in which higher amounts of carvacrol accounted for higher antibacterial efficacy than geraniol [64, 65]. The similar inhibitory concentrations of *T*. *longicaulis* EO as presented by De Martino *et al*. [66] disprove the importance of the ratio between oxygenated monoterpenes and monoterpene hydrocarbons. This implies that, beside the fact of using different classification for phenols, the variety of synergistic and additional modes of antimicrobial action can be related to both inactive and weakly active compounds in different quantitative proportions.

There is a limited amount of reports on the antibiofilm activity of the tested plant species [61, 62, 67, 68]. Two main components of *S*. *sclarea*, namely salvipisone and aethiopinone, effectively reduced biofilm volume produced by *S*. *aureus* and *S*. *epidermidis* [61, 62]. Since intraoral bacterial glucosyltransferases (GTases) synthesize main extracellular matrix substrates such as glucan and fructan from sucrose contributing to the bacterial biofilm formation on tooth surfaces, the enhanced inhibitory activity of aqueous and methanol extract of rosemary against GTase of *S*. *sobrinus* is noteworthy [67]. This was confirmed by Quave *et al*. [68] with an ethanol extract, which reduced biofilm production of methicillin-resistant *S*. *aureus*. Even *P*. *aeruginosa* was eradicated by the EO of *O*. *vulgare* spp. Interestingly, in the present study although the OD values of the extracts were generally more scattered compared to the OD values of CHX, extract concentrations ≥0.3 mg ml^-1^ had moderate to high antibiofilm activity against *S*. *mutans*.

In conclusion, all tested Mediterranean herbs inhibited effectively the screened obligate anaerobic oral microorganisms. Thus, the null hypothesis was rejected. The outcome of this microbiological screening encourages further investigations to encounter and develop natural components with antimicrobial and antibiofilm activity against oral pathogens. Due to the increasing incidence of multi-resistant bacteria despite the use of oral disinfectants e.g. chlorhexidine, the application of herb extracts could prove to be an effective alternative treatment strategy against oral pathogens [69, 70]. Interestingly, diverse side effects of conventional oral care products such as allergies, intolerable taste, tooth coloring, toxicity, and antimicrobial resistance have triggered the search for alternative, in best case natural, antimicrobials. The development of natural resources is crucial for developing countries contributing to economic growth and enhancing people’s health at low cost [22, 60, 71, 72]. Overall, methanol extracts from *R*. *officinalis* and *S*. *sclarea* had the most significant antimicrobial effects against all tested oral pathogens, while *Cistus* spp. extracts exhibited the highest antibiofilm activity against *S*. *mutans*. Thus, combinations of these plant extracts could serve as main antimicrobial components in alternative antibacterial formulations facilitating the prevention of biofilm-related oral diseases such as caries or periodontitis.

## Abbreviations

CBA: Columbia blood agar plates
CFU: colony forming units
CHX: chlorhexidine
CLSI: Clinical and Laboratory Standards Institute
DSM: German Collection of Microorganisms and Cell Cultures
EO: essential oil
GTase: glucosyltransferase
HCB: yeast-cysteine blood agar plates
MBC: minimal bactericidal concentration
MHB: BBL Mueller Hinton II Broth-Cation-Adjusted
MHB-S: MHB containing 1% saccharose
MIC: minimal inhibitory concentration
OD: optical density
PBP: penicillin binding protein
QS: quorum sensing
UAE: ultrasound assisted extraction
WCB: Wilkins-Chalgren broth
